# Retraction: A novel tumor suppressor gene, ZFN24, inhibits the development of NSCLC by inhibiting the Wnt signaling pathway to induce cell senescence

**DOI:** 10.3389/fonc.2022.1045516

**Published:** 2022-09-27

**Authors:** Frontiers Editorial Office

**Keywords:** ZNF24, non-small cell lung cancer (NSCLC), WNT signaling pathway, senescence, tumor suppressor genes (TSGs)

The Publisher retracts the cited article.

Following publication, concerns were raised regarding the authorization and misinterpretation of some data used in this study. Specifically, during the investigation, which was conducted in accordance with Frontiers’ policies, the authors failed to provide appropriate authorization for use of some of the original data. In addition, the Chief Editors of Frontiers in Oncology concluded that the article’s conclusions were not sufficiently supported by the findings from the data provided. Given that unauthorized data use is a breach of Frontiers’ guidelines, and that the Chief Editors expressed concern about the soundness of the conclusions, the article is being retracted.

This retraction was approved by the Chief Editors of Frontiers in Oncology and the Chief Executive Editor of Frontiers. The authors and authors’ institution agree to this retraction.

